# Characteristics of Polyaniline Cobalt Supported Catalysts for Epoxidation Reactions

**DOI:** 10.1155/2014/648949

**Published:** 2014-02-18

**Authors:** Grzegorz Kowalski, Jan Pielichowski, Mirosław Grzesik

**Affiliations:** ^1^Faculty of Food Technology, University of Agriculture in Krakow, Balicka 122, 30-149 Kraków, Poland; ^2^Department of Polymer Science and Technology, Cracow University of Technology, Warszawska 24, 31-155 Kraków, Poland; ^3^Institute of Chemical Engineering, Polish Academy of Science, ul. Bałtycka 5, 44-100 Gliwice, Poland

## Abstract

A study of polyaniline (PANI) doping with various cobalt compounds, that is, cobalt(II) chloride, cobalt(II) acetate, and cobalt(II) salen, is presented. The catalysts were prepared by depositing cobalt compounds onto the polymer surface. PANI powders containing cobalt ions were obtained by one- or two-step method suspending PANI in the following acetonitrile/acetic acid solution or acetonitrile and then acetic acid solution. Moreover different ratios of Co(II) : PANI were studied. Catalysts obtained with both methods and at all ratios were investigated using various techniques including AAS and XPS spectroscopy. The optimum conditions for preparation of PANI/Co catalysts were established. Catalytic activity of polyaniline cobalt(II) supported catalysts was tested in dec-1-ene epoxidation with molecular oxygen at room temperature. The relationship between the amount of cobalt species, measured with both AAS and XPS techniques, and the activity of PANI-Co catalysts has been established.

## 1. Introduction

Heterogeneous polymer supported catalysts, in particular on conjugated polymers, have been extensively studied in the last few years [1–5]. This class of polymers has an extended *π*-conjugated bond system in its polymer backbone and is therefore capable of conducting electricity. One of the most intensively studied conjugated polymers is polyaniline (PANI) because of its low price, availability, easy synthesis route, resistance to variety of reaction conditions, and interesting redox properties related to the nitrogen atom present in its polymer backbone. Also, it should be noted that incorporation of transition metal anions under appropriate conditions can be achieved by means of a doping reaction. That way polymer and catalytically active metals can form PANI-transition metal complexes, which are stable in the reaction medium and, due to the insolubility of PANI in common organic solvents, the catalyst could be easily recycled. Different transition metal systems have been introduced into the polyaniline matrix such as cobalt [1, 6–10], copper [11–13], platinum [14–16], or palladium [17–19] which were used in form of ions or metal complexes. PANI-transition metal systems cover different kinds of organic transformations such as hydrogenation [19–22] or oxidation reactions [1, 8–10, 18, 23, 24].

Many oxidation processes are characterized by low selectivity, which makes them much more difficult in application [6, 7, 25–28]. Heterogeneous oxidation of organic compounds can proceed selectively and efficiently with a wide range of organic compounds and the typical polymer is very stable in an oxidative atmosphere [29]. Such phenomenon is observed due to the fact that the doping reaction with use of transition metal salts or complexes in appropriate conditions modifies the electronic properties of PANI, and therefore it is able to transport electrons. Protonic acid doping converts a semiconducting emeraldine base to the conductive derivative. In our previous paper it was demonstrated that polyaniline (PANI) supported by cobalt or cobalt complexes serve as a synthetic metal catalyst in the oxidation of different varieties of alkenes [8–10]. Some characteristics of Co(II)-PANI based catalysts were studied in our previous article [8]. More detailed studies of CoCl_2_ doped catalysts synthesized in HCl or LiCl solutions were also presented [3]. However, according to our best knowledge, preparation and characterisation of catalysts based on different Co(II) salts or complexes synthesized in different conditions have not been investigated so far.

In this study, various Co(II) salts, that is, cobalt(II) chloride, cobalt(II) acetate, and cobalt(II) salen complex, have been selected. Various doping methods and Co(II) : PANI ratios were used to obtain PANI-Co powders. Physicochemical properties determined by AAS and XPS spectroscopy have made it possible to draw conclusions on the structure of cobalt ion binding. These catalysts have been investigated and tested in dec-1-ene epoxidation. The catalytic activity data of the obtained catalysts were correlated with the doping method, Co(II) compound used in PANI-Co system, and finally cobalt amount which was determined by using atomic absorption spectroscopy (AAS) and X-ray photoelectron spectroscopy (XPS). The work continues our research on synthesis, characterization, and testing of oxidation reactions in polyaniline supported cobalt(II) catalysts [8–10].

## 2. Experimental

Polyaniline was obtained via an oxidative polymerization method [9, 30]. Cobalt(II) salts, for example, cobalt(II) acetate and cobalt(II) chloride, were of analytical grade. Co(II) salen have been synthesized as described [31]. All salts and complexes were immobilized on PANI as described previously [9]. A series of polyaniline supported cobalt(II) based catalysts were prepared by the following procedures. All samples were presented at Table 1.

### 2.1. Polyaniline Supported Catalysts Synthesis: Method I

A mixture of polyaniline (500 mg) and cobalt acetate (500 mg) was stirred in an MeCN (25 mL) and HOAc (25 mL) mixture for 72 h at r.t. Then the reaction mixture was filtered and the solid catalyst was washed with MeCN (5 × 5 mL). The catalyst was dried at 110°C for 24 h.

### 2.2. Polyaniline Supported Catalysts Synthesis: Method II

A mixture of polyaniline (500 mg) and cobalt acetate (500 mg) was stirred in an MeCN (50 mL) mixture for 72 h at r.t. Then the reaction mixture was filtered and dried. Then catalyst was stirred in an AcOH (50 mL) for 1 h at r.t. The solid catalyst was washed with MeCN (5 × 5 mL). The catalyst was dried at 110°C for 24 h.

The amount of cobalt introduced into PANI was determined by atomic absorption spectrometry (AAS) in Perkin Elmer AAnalyst 300 spectrometer after dissolution of the PANI-Co samples in HNO_3_.

Surface analysis of the catalyst was made with the XPS method in a VSW 100 spectrometer using Mg *K*_*a*_ radiation (1253,6 eV). The operating pressure was 3 × 10^−6^ Pa. The catalyst powdered samples were mounted on double-sided tape. The following routines were applied for data acquisition and analysis: a standard method for deconvolution using a mixed Gaussian-Lorentzian line shape always in the same proportion, 20% Lorentzian and 80% Gaussian. The position of partial peaks as well as full width at the maximum was kept constant. An energy correction was made to account for sample charging based on the C 1*s* peak at 284.6 eV as the inert standard. The surface composition was determined using sensitivity factors. The fractional concentration of a particular element *A* (%*A*) was computed using

(1) $$\begin{array}{c} x_{i_{\text{surface}}}=\frac{I_{i}/S_{i}}{\sum \left(I_{i}/S_{\begin{array}{c} i \end{array}}\right)}, \end{array}$$

where *I*_*i*_ and *S*_*i*_ are integrated peak areas and the sensitivity factors, respectively.

Catalytic activity of our catalysts was controlled on epoxidation of dec-1-ene according to the method described previously [9].

## 3. Results and Discussion

Taking into consideration content of the cobalt atoms on the surface of the tested catalysts and the total amount based on AAS, in relation to the number of nitrogen atoms, it is clearly evident that the concentration at the surface is at least equal to or in some cases 7.13 times higher than the concentration of cobalt measured in the whole volume of the sample (Table 2). Therefore, an important conclusion can be drawn that immobilized small molecule compound is focused mainly on the polymer surface.

Furthermore, the ratio of Co : N atoms for catalysts based on CoCl_2_ (168A and 168B) is much higher than that for the other catalysts, indicating that in this case more nitrogen atoms are involved in the cobalt-nitrogen bond formation. In the case of catalysts based on cobalt acetate(II) (169A, 170B) saturating the nitrogen atoms with cobalt ones is several times lower and ranges from 0.018 to 0.027 Co atoms per nitrogen atom. In the case of catalysts 169B and 170A, surface concentration of Co was too low to give detectable spectral lines for cobalt. The catalyst based on Co(II) salen (159A) does not show the presence of cobalt spectral lines.

Stoichiometric variation of carbon atoms was observed in some samples from the value expected for the ideal PANI structure. This phenomenon is probably due to a significant amount of oxygen adsorbed in the form of water, which is very difficult to remove from the polyaniline surface. As it was presented in literature water molecules are present even in the dried samples [32].

**Figure 1:**
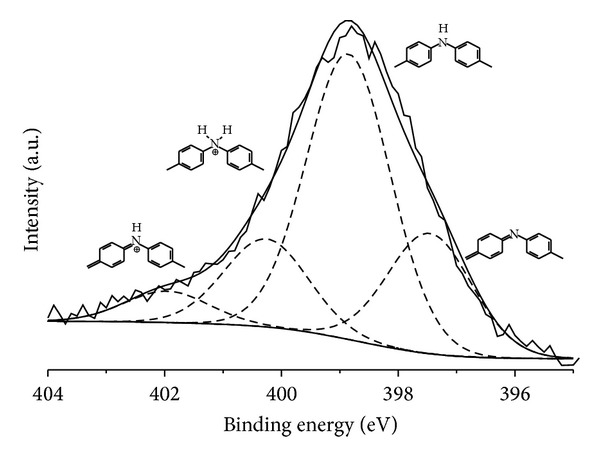
Representative N1*s* spectra of polyaniline cobalt(II) supported catalyst.

Detailed analysis of the XPS spectra allows for a more precise determination of nature of the chemical bonds on the surface. According to literature reports [33–35] the N1s spectra of polyaniline can be described with four components at 398.20, 399.40, 400.7, and 402.6 eV, which can be assigned to different polyaniline units: quinonoid, benzenoid, protonated benzenoid, and protonated quinonoid, respectively (Figure 1).

**Figure 2:**
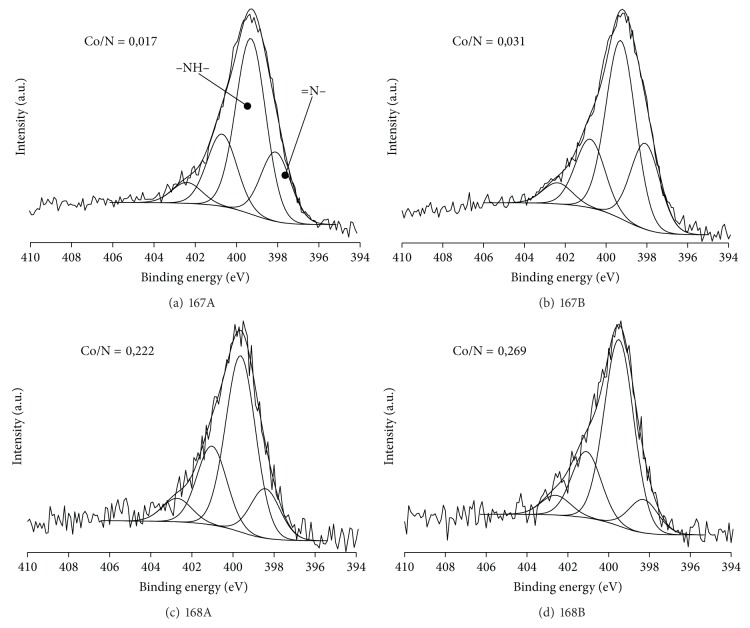
XPS N1*s* spectra for CoCl_2_ immobilized on polyaniline (167A-168B).

**Figure 3:**
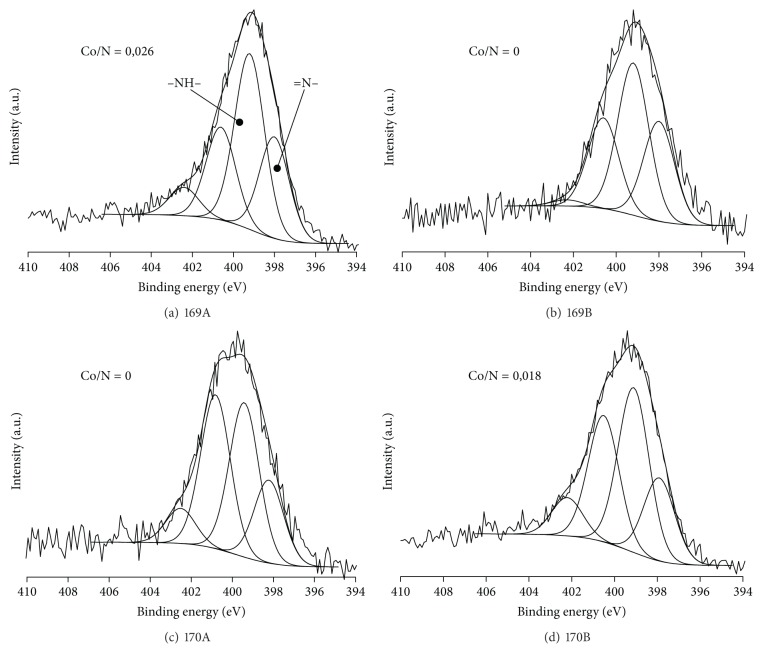
XPS N 1*s* spectra for cobalt(II) acetate immobilized on polyaniline (169A-170B).

Figures 2(a)–2(d) and Figures 3(a)–3(d) show N1*s* spectra of polyaniline supported catalysts for the cobalt(II) chloride and cobalt(II) where different ratios of cobalt salt to polymer and different doping conditions were used. However, Table 3 shows exact values for all components of nitrogen spectra. A decrease in the degree of oxidation of the polyaniline chain was observed with an increasing of Co content on the surface, by reducing the quinonoid units to benzenoid ones. Analyzing the N1*s* spectra, the increasing in band associated with benzenoid units (indicated as –NH– in Figure 2) is visible as is the simultaneous decrease of the quinonoid unit band. At the same time there was no change observed in the protonation degree of polyaniline or in the ratio of protonated quinonoid and benzenoid units. It could be concluded that in the polyaniline doping reaction with cobalt compounds unprotonated polyaniline units were involved.

The decline in the oxidation state of the polymer, as a result of doping with cobalt salts, suggests that Co atoms interact more strongly with the nitrogen atom of the quinonoid units than with amine ones included in the benzenoid units. Theoretical considerations also point to the fact that the imine groups of PANI are much more reactive [36]. It should also be noted that in polymer doped with cobalt salts (II) the whole N1*s* band is shifted for 0.2–0.5 eV to higher binding energy range. Moreover shift increases with increasing amount of Co in the catalyst. This phenomenon could be explained by the charge transfer from the nitrogen atom of PANI to the cobalt atom, which results in binding energy increasing. It can be concluded that there is a chemical interaction between cobalt atoms and nitrogen atoms of the polymer. It was clearly visible for catalysts based on CoCl_2_ (167A-168B). Doping with cobalt acetate is not as effective; therefore taking into account XPS method restrictions, it is not possible to provide observations of these changes. However, a slight shift (+0.15 eV) can be observed for 169A, in comparison with catalysts without cobalt.

There were no significant changes in protonated units content for CoCl_2_ based catalysts (167A-168B) with increasing of cobalt amount in the catalyst, with the protonated units content remaining at a relatively low level. The situation is completely different for the catalysts in which the polyaniline is doped with cobalt(II) acetate (169A-170B). Protonation degree for these catalysts is much higher when compare with PANI. Lower protonation degree was observed for catalyst 169A only, for which the ratio of Co/N was 0.0258. Thus, for catalysts which contain measurable amounts of cobalt, protonation is negligible due to blocking the nitrogen atoms with cobalt ones. This is another proof of the existence of the chemical nature of the interaction between nitrogen and cobalt atoms immobilized on polyaniline.

**Figure 4:**
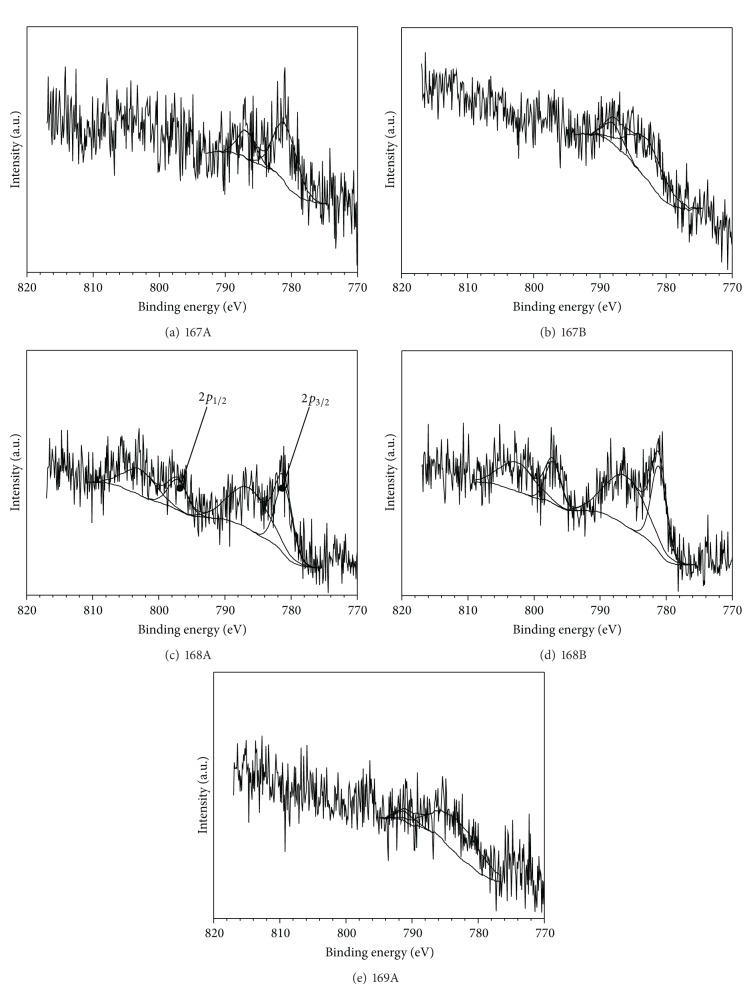
XPS Co 2*p* spectra for cobalt(II) acetate and CoCl_2_ immobilized on polyaniline.

Cobalt 2*p* spectral lines are similar for all catalysts with detectable amounts of cobalt atoms (Figure 4). Binding energies could be assigned according to the data presented in the literature [37–39]. Binding energies for the two possible spin states 781.2 eV and 797.5 eV are responsible for 2*p*_3/2_ and 2*p*_1/2_ transitions, respectively. Small shifts depending on the catalyst were observed. Furthermore, in Co2*p* spectra, one satellite peak appears for each 2*p* transition. The signal-to-noise ratio was too small to properly assign the appropriate mathematical function.

Based on the 2*p*_3/2_ peak position it was virtually impossible to get any information on the chemical environment. Most information can be obtained by analyzing the gap between 2*p*_3/2_ and 2*p*_1/2_ peaks, their relative intensity, and structure of the satellite bands. The presence of strong satellite bands for all catalysts indicates that the degree of oxidation of the cobalt atom during immobilizing on PANI was not changed and is equal to +2. Moreover Co(II) has an octahedral structure and a high-spin state. According to data in the literature, cobalt compounds in which the oxidation state is equal to +3 have very weak satellite peaks or do not have them at all [40]. In addition, the literature showed that by analyzing the ratio of satellite peak intensity to the intensity of main peak (*I*_sat⁡_/*I*_*M*_) and the energy difference between them, one can get information about the nature of the metal binding ligand [37]. Namely, when the difference in energy increases and the ratio of the satellite to the main peak decreases then the covalent nature of the metal-ligand bond is increasing (Table 4). It appears that for catalysts 168A and 168B, which have a much higher Co content in comparison to the other catalysts, the intensity ratio of satellite to main peak is much higher. Therefore, with increase of cobalt content in the catalyst, increasing of covalent bonding nature of cobalt was observed.

**Figure 5:**
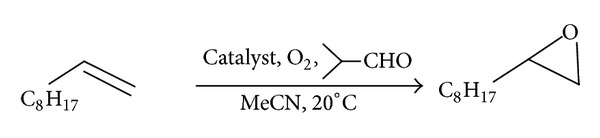
Reaction of dec-1-en epoxidation.

The catalytic activity of the obtained catalysts was tested in the epoxidation of dec-1-ene (Figure 5). The results of the epoxidation reaction, combined with the Co content determined by AAS and XPS, were presented at Table 5.

Comparing catalysts synthesized in the same conditions and with the same substrates but with a different content of Co (taking into account the content of Co on the surface), it could be observed that the reaction yield is increasing with an increase of Co amount. Comparing Co content determined by AAS and XPS, it was observed that for catalyst 167A the cobalt amount determined using AAS is higher than in 167B and is equal to 0.0175 and 0.0162 mol of Co/mol of N, respectively. While XPS analysis for catalysts 167A and 167B was given completely different results—0.0172 and 0.031 mol of Co/mol of N. It follows that part of the cobalt was trapped inside polymer clusters. Moreover, taking into account the efficiency of the epoxidation reaction, which increases with increasing of surface concentration of Co (for the same conditions of catalyst synthesis), it can be concluded that in epoxidation reaction only cobalt compounds located on the catalyst surface were involved, while the part of the Co trapped inside polymeric clusters was inactive in the epoxidation reaction.

It was also observed that the epoxidation with use of the catalysts synthesized by a two-step method occurs with higher yields than the corresponding catalysts synthesized by one-step method. The presence of a relatively strong acid in reaction media during immobilization results in the protonation of polyaniline and the blocking of the free electron pair, which may act as a potential electron donor to the cobalt atom. Such a phenomenon is observed in the case of doping with cobalt(II) acetate, where the protonation degree was at the level of 40–45% for the one-step method and 28–30% for the two-step method (Table 3).

## 4. Conclusions

A series of novel conductive polymer supported cobalt catalysts based on polyaniline and cobalt(II) compounds (cobalt(II) chloride, cobalt(II) acetate, and cobalt(II) salen) have been developed. Investigations of incorporation of Co(II) ions into polyaniline together with the studies of physicochemical properties of PANI-Co systems have shown the following.

1. Properties of catalysts strongly depend on method of cobalt(II) immobilization on the polymer matrix.
2. Comparing results from AAS and XPS analysis, it may be concluded that immobilized cobalt based molecules are located mainly on the polymer surface.
3. Some steric hindrance is observed when large molecules were used as doping agents. The largest effective immobilization was when CoCl_2_ was used.
4. Doping reactions occur mainly on unprotonated polyaniline units. Some charge transfer from the nitrogen atom of PANI to the cobalt atom was observed for catalysts 167A-168B.

## Figures and Tables

**Table 1:** Representation of synthesized polyaniline supported catalysts.

	Co(II) source	Doping method	Catalyst/PANI (g/g)
159A	Co(II) salen	II	1 : 1

167A	CoCl_2_	I	1 : 2
167B		I	2 : 1
168A		II	1 : 2
168B		II	2 : 1

169A	Co(CH_3_COO)_2_·4H_2_O	I	1 : 2
169B		I	2 : 1
170A		II	1 : 2
170B		II	2 : 1

**Table 2:** Comparing results obtained from AAS analysis and semiquantitative chemical analysis obtained from X-ray photoelectron spectroscopy.

Sample	XPS (surface)					AAS (bulk) Co/N	Co_XPS_/Co_AAS_
	C	N	Co	Cl	O		
154	8.68	1		—	0.73	0.0000	—
159A	13.32	1		—	1.51	0.0039	—
167A	7.01	1	**0.017**	0.24	0.45	0.0175	**0.98**
167B	8.35	1	**0.031**	0.25	0.86	0.0162	**1.89**
168A	9.93	1	**0.222**	1.02	1.18	0.0889	**2.50**
168B	9.74	1	**0.269**	1.48	0.92	0.1065	**2.52**
169A	8.54	1	**0.027**	—	0.87	0.0105	**2.46**
169B	9.28	1		—	0.97	0.0153	—
170A	10.09	1		—	1.03	0.0146	—
170B	11.13	1	**0.018**	—	1.51	0.0097	—

*The content of the individual atoms both XPS and AAS measurements were normalized to nitrogen.

**Table 3:** Binding energies of nitrogen N1*s*.

Sample	Binding energy (eV)				Peak contribution (%)			
	–N=	–NH–	–NH_2_^+^–	–NH^+^=	–N=	–NH–	–NH_2_^+^–	–NH^+^=
PANI	397.9	399.3	400.7	402.4	22.9	54.3	16.8	6.0
159A	398.0	399.4	400.8	402.5	14.4	33.5	35.3	16.7
167A	398.1	399.3	400.7	402.4	51.9	20.5	21.4	6.2
167B	398.2	399.4	400.9	402.5	50.3	24.3	19.4	6.0
168A	398.4	399.6	401.0	402.7	53.7	15.1	24.0	7.2
168B	398.3	399.5	401.1	402.6	61.0	10.9	21.7	6.4
169A	398.0	399.2	400.6	402.4	25.5	44.1	23.3	7.1
169B	398.0	399.2	400.6	402.3	28.6	43.6	26.0	1.8
170A	398.3	399.5	400.9	402.6	19.5	36.2	36.1	8.2
170B	397.9	399.1	400.5	402.2	20.1	39.9	30.6	9.3

**Table 4:** Characteristic values in Co 2*p* spectra for catalysts presented in Figure 4.

Sample	Co 2*p*_3/2_/eV	Satellite/eV	*I*_sat⁡_/*I*_*M*_	Co 2*p*_1/2_/eV	Satellite/eV	*I*_sat⁡_/*I*_*M*_	Δ[Co 2*p*_1/2_ − Co 2*p*_3/2_]/eV
167A	781.1	786.9	0.33	—	—	—	—
167B	783.0	788.1	0.29	—	—	—	—
168A	781.5	786.7	1.41	797.5	803.2	1.38	16.0
168B	781.4	786.4	1.57	797.4	802.9	1.56	16.0
169A	783.5	791.4	0.095	—	—	—	—

*I*_sat⁡_/*I*_*M*_: intensity ratio of satellite to fundamental band.

**Table 5:** Dec-1-ene epoxidation on PANI Co(II) supported catalysts (reaction time 48 h).

	Co (AAS)/(mol_Co_/mol_N_)	Co (XPS)*/(mol_Co_/mol_N_)	Yield/%
159A	0.0039	—	38.0
167A	0.0175	0.0172	39.3
167B	0.0162	0.031	57.3
168A	0.0889	0.222	22.8
168B	0.1065	0.269	26.1
169A	0.0105	0.0258	44.7
169B	0.0153	—	41.8
170A	0.0146	—	26.0
170B	0.0097	0.018	26.8

*Co content in catalyst 159A. 169B and 170A were below the sensitivity of the XPS method.
